# Near Work Related Behaviors Associated with Myopic Shifts among Primary School Students in the Jiading District of Shanghai: A School-Based One-Year Cohort Study

**DOI:** 10.1371/journal.pone.0154671

**Published:** 2016-05-03

**Authors:** Xiaofang You, Ling Wang, Hui Tan, Xiangui He, Xiaomei Qu, Huijing Shi, Jianfeng Zhu, Haidong Zou

**Affiliations:** 1 Department of Maternal and Child Health, School of Public Health, Key Laboratory of Public Health Safety, Ministry of Education, Fudan University, Shanghai, China; 2 Department of Eye Disease Prevention, Shanghai Eye Disease Prevention & Treatment Center, Shanghai, China; 3 Eye & ENT Hospital, Fudan University, Shanghai, China; Rush University Medical Center, UNITED STATES

## Abstract

**Purpose:**

To investigate the characteristics of various near work related behaviors among primary students and their associations with changes in myopia related ocular biometric parameters during one-year of follow up.

**Methods:**

A school-based sample of 4,814 primary 1^st^ to 4^th^ grade students aged 6–10 years old were selected by cluster randomization based on probability proportion to size in 2013. At baseline, students together with their parents filled in a self-administered questionnaire on 9 aspects of near work related behaviors and some important covariants of myopia. A comprehensive set of eye examinations including axial length (AL) and cycloplegic refraction was conducted both at baseline and one year later.

**Results:**

With the grade level increase, students did increasingly better at finding various ways to have an eye break, but they were increasingly likely to continuously do long-time near work without an eye break. Keeping a reasonable eye distance and correct hand posture for reading, writing, or watching TV became worse for the first time before grade 2, but then became better at grade 3. In contrast, selecting appropriate lighting environments or situations and keeping a balanced diet became better for the first time before grade 2, but then became worse at grade 3. At one-year follow up, the mean AL increased by 0.32 ± 0.35 mm, the ratio of AL divided by the mean corneal radius of curvature (AL/CR ratio) increased by 0.032 ± 0.054, the myopic spherical equivalent (SE) increased by -0.51 ± 0.51 diopters and the incidence of myopia was 16.0% (237/1,477). After controlling for the confounding effects of parental myopia, student’s age, gender, height, daily near work time, daily outdoor activity time and all of the other near work related behaviors, keeping a reasonable distance when reading, writing and watching TV was associated with elongation of the AL [standard coefficient beta = -0.062, *P* = 0.004], a change in SE [beta = -0.072, *P* = 0.020] and incident myopia [adjusted odds ratio (aOR) = 0.90, 95% confidence interval (CI): 0.84–0.96]. Selecting an environment with adequate light for visual comfort to read and write was related to elongation of the AL [beta = -0.039, *P* = 0.034] and increase of AL/CR ratio [beta = -0.030, *P* = 0.048]. Also, not continuing to do near work for more than 30–40 minutes without an eye break was related to increase of the AL/CR ratio [beta = -0.028, *P* = 0.044] and a change in SE [beta = -0.064, *P* = 0.023].

**Conclusion:**

Various near work related behaviors changed according to grade level in primary school students. Independent of hereditary factors, daily near work load and outdoor activity, near work related behaviors such as keeping an inappropriate eye distance for near work, selecting inadequate lighting environments, and continuing to do near work without an eye break were risk factors for myopic shifts.

## Introduction

Myopic change is becoming a global public health problem with high costs associated with correction and treatment for complications such as myopic macular degeneration, retinal detachment, glaucoma, and cataract [1]. According to a study from Singapore, the mean annual direct cost of myopia for each Singaporean school child aged 7–9 years was estimated to be US $148[2]. Based on the world-wide prevalence of myopia, in countries in east and southeast Asia, such as Singapore, China, Taiwan, Hong Kong, Japan, and Korea, the prevalence of myopia has rapidly increased in the past 50–60 years [3]. In China, the prevalence of myopia is tremendously high and up to 90% of teenagers and young adults have myopia [4]. According to The Anyang Childhood Eye Study, the rate of myopia was 3.9% in grade 1 and 67.3% in grade 7 [5].

Factors identified as associated with myopia included parental myopia [6,7], outdoor time [8,9,10] and near work time [8,9]. However, the association between near work time and myopia was inconclusive. Several studies, including The Sydney Adolescent Vascular and Eye Study (SAVES) and The Xichang Pediatric Refractive Error Study (X-PRES), reported no association between time spent on near work activities and myopia [8,9,11]. Some studies indicated that near work times were associated with myopia [7,8]. Ip et al. reported myopia was not associated with time spent doing near work, but with close reading distance and continuous reading [12]. It seems that near work related behaviors had a confounding effect on the relation between near work time and myopia. Limited studies have explored some special near work related behaviors such as reading distance and reading illumination and their associations with myopia [7,13,14]. Yang et al. reported reading distance affected accommodative responses of students [13,14], and You and colleagues found dim reading illumination was related to a higher prevalence of myopia [7]. However, these studies focused on one or several behaviors or environments and few studies have fully explored the comprehensive and multi-dimensional aspects of near work related behaviors. What is more, the outcome used to reflect myopia was mainly noncycloplegic autorefraction, which has relatively high sensitivity and specificity for detecting myopia in older children, but is likely to have led to a progressively greater proportion of nonmyopic subjects being classified wrongly as myopic at earlier ages, due to the tendency for younger subjects to accommodate more during the test [15]. Moreover, limited studies have used a longitudinal design to explore the process of myopia progression under accurate observation, and provided causative clues for the prevention and treatment of myopia.

This study was intended to comprehensively and multi-dimensionally explore characteristics of near work related behaviors in primary school students in Shanghai, China and their associations with myopia related oculometric parameters, including both biometry indicators such as axial length (AL) and the axial length-corneal radius (AL/CR ratio), and indicators after cycloplegic refraction, such as spherical equivalent (SE) and incident myopia. To fully understand the causal effects of near work related behaviors on myopia, we analyzed the data of near work related behaviors at baseline (before the progression period) and their associations with myopia progression through a prospective study design.

## Methods

### Study design

The study was part of a 3-year public health program (2011–2013) to establish archives of the refractive status of children in Shanghai, which was aimed to describe the prevalence of myopia, analyze factors related to myopia and develop personalized assessment tools, develop parameters of refractive development for preschool students and primary school students, draw age specific curves and determine cut-off values for myopia screening and establish three levels of preventive networks for myopia. The project was conducted by the Department of Maternal and Child Health of the School of Public Health of Fudan University, Department of Eye Disease Prevention, Shanghai Eye Disease Prevention and Treatment Center, and the Eye and ENT Hospital of Fudan University from September 2013 to October 2014. The design was a school-based one-year cohort study.

### Study participants

The data were from the Elaborative Shanghai Childhood Ocular Refractive Development Study (E-SCORDS) in China. This study was focused on the near work related behaviors and their associations with myopic shifts in primary school students in the Jiading District of Shanghai, China. Based on research by He and colleagues in Guangzhou, the 5-year cumulative incidence of myopia in 6-year-old children was calculated to be 25.7%, and the 2-year cumulative incidence of myopia in 12-year-old children was 31.4% [16]. Sample size was based on estimating the one-year incidence of myopia at 25.7%, with a limit of ±5% for the 95% confidence interval (CI; α = 0.05, d = 0.1p). Considering a design effect of 1.5, nonparticipation of 20% and a loss of follow up of 20%, 2,604 students were required. According to the data provided by the education sector of Jiading district in 2013, there were 33,097 schoolchildren aged 6 to 13 years old in grades from 1 to 5 in 34 primary schools. A cluster randomization based on probability proportion to size was used. Seven primary schools including 5,826 students in grades from 1 to 5 were randomly selected. As the study intended to follow the students for one year to explore near work related behaviors associated with myopic shifts, we only included students in grades 1 to 4 (4,814 of 5,826) to reduce the rate of loss to follow-up.

**Fig 1:**
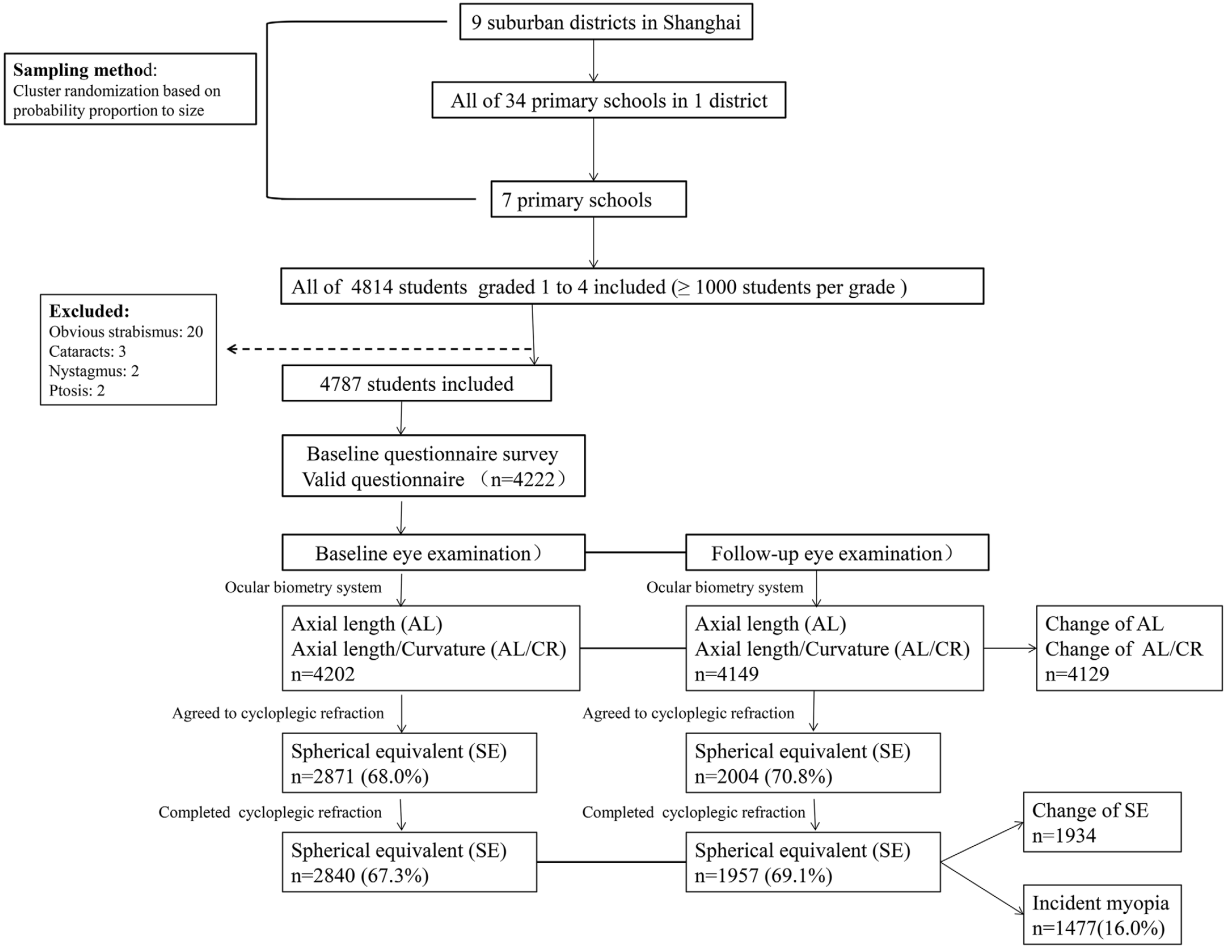
Flow chart of participant recruitment.

At baseline (2013), with exclusion of the cases with obvious strabismus (20), cataracts (3), nystagmus (2), and ptosis (2), 4,787 of the 4,814 sampled and registered students were included in the analysis. Among the 4,787 that were planned to complete the questionnaire survey, 4,222 (88.2%) finished the questionnaire regarding basic information, near work time, outdoor activities, and near work related behaviors. Of the 4,222 completed questionnaires, 2,871 (68.0%) agreed to undergo all the examinations including cycloplegic refraction. On the day the examinations were performed, the actual number of students who finished participating in the examinations was 2,840 (67.3%). In 2014, 2,832 (99.7%) of 2,840 students examined at baseline returned for the follow-up examination, whereas only 2,004 (70.8%) students agreed to have cycloplegic refraction, and 1,957 (69.1%) finished cycloplegic refraction (Fig 1).

### Examination

Eye examinations were performed by a clinical team composed of 5 optometrists, 2 public health doctors, 1 ophthalmologist, 1 field assistant, 1 school nurse, and 1 study manager. An experienced public health physician specializing in the prevention of children’s eye diseases from the Shanghai Eye Disease Prevention and Treatment Center was made the project coordinator and ran the entire investigation. All children completed a comprehensive eye examination at both baseline and one-year follow-up.

The examination process began with testing visual acuity at 4 m using a retro-illuminated Early Treatment of Diabetic Retinopathy Study (ETDRS) chart with tumbling-Eoptotypes. Acuity was tested with and without refractive correction for those wearing spectacles. Cycloplegic refraction was performed with a Desktop Autorefractor (Model No.: KR-8800; Topcon Corporation, Tokyo, Japan) with a measurement range of -20 to +20 diopters (D). This instrument provided a median value of 3 reliable readings in each eye. The medians were used for the analysis. Each student was reexamined if the differences between the any 2 results of the 3 results obtained were greater than 0.50 D. Cycloplegia was induced in each eye by the instillation of 1% cyclopentolate. Each student was first administered one drop of topical anesthetic agent (Alcaine, Alcon) to alleviate discomfort, followed by one drop of 1% cyclopentolate (Alcon) after a 15-second interval. One more drop 1% cyclopentolate (Alcon) was given after 5 minutes. Twenty minutes after the last drop, a third drop of cyclopentolate was administered if the pupillary light reflex was still present or the pupil size was less than 6.0 mm [17]. Measurements of ocular biometric parameters (axial length and keratometry) were performed with an ocular biometry system (IOLMaster; version 5.02, Carl Zeiss Meditec, Oberkochen, Germany). The subjects were required to blink prior to measurement to make sure the tear film covered the whole cornea. The measurements of AL were considered valid if individual measurements varied by no more than 0.02 mm. Any students presenting visual acuity not better than 20/25 in either eye were given subjective optometry to obtain the best corrected visual acuity. All cases in which results could not be obtained and in which there were large differences between examination and reexamination values were recorded in the remarks. Anterior segments and the fundus were examined with a slit-lamp and ophthalmoscope, and abnormalities in eyelids, conjunctiva, cornea, lens, vitreous fluid, and fundus were recorded. Strabismus examination was performed using cover testing.

### Questionnaire survey

The survey was divided into two phases: the school mobilization and questionnaire interview phases. School mobilization was implemented in selected school sampling units by project members and school teachers through a meeting in which the details regarding the questionnaire were explained to the parents and guardians. The mobilization was conducted two to three days before the questionnaire survey. Together with class teachers, the project members gave the students the questionnaire and gave them detailed instructions on how to fill it in with their parents or guardians. Students’ parents or guardians, together with the students, answered the self-administered questionnaire with detailed instructions at home and gave it to the class teachers on the next day. An audit of the questionnaire was conducted to increase its validity, with verification procedures that included checks by class teachers, audits by quality control staff (senior post-graduate students from the School of Public Health of Fudan University), and phone reviews when necessary.

A detailed questionnaire was administered to obtain information on students’ demographic characteristics, students’ activities, and parental myopia at baseline. Students’ activities included near work activities, outdoor activities, and near work related behaviors. Near-work activities were mainly focused on homework-based time (such as exercises or tutoring), and paper-based reading time (such as books, magazines and so on). The assessment of outdoor time was based on questions about walking, playing outdoors, having picnics, and playing outdoor sports. The above information was obtained by inquiring about the average time spent on such activities during weekdays and weekends separately. The average number of daily near work and outdoor activity hours was calculated using the following formula: [(hours spent on a weekday) × 5 + (hours spent on a weekend day) × 2] / 7. A parent was considered as myopic if he or she used spectacles primarily for distance or for both distance and near.

Questions about students’ near work related behaviors in the “last” week were taken directly from the Child Vision Care Behaviors Scale (CVCBS) (S1 File)[18] and covered nine subscales: finding various ways to take eye breaks (7 items), not continuing to do near work for more than 30–40 minutes without an eye break (3 items), doing eye exercises according to the right way (2 items), keeping a reasonable distance when reading, writing and watching TV (5 items), keeping a correct hand posture to hold a pen when writing (2 items), keeping correct body posture when reading and writing (3 items), not reading in situations that can lead to an unstable body posture (6 items), selecting an adequate lighting environment for visual comfort when reading and writing (2 items) and keeping a balanced diet (2 items). By asking “How often do you have the special behavior?”, the response to each item was coded as “never,” “occasionally,” “often” and “always” on a Likert scale of 1 to 4, respectively. The total score was equal to the 32 item scores added together, and total scores ranged from 0 to 128. The students with higher scores meant they had better behaviors [18]. The scale was primarily tested to determine its validity and reliability. The Cronbach’s α coefficient was 0.842, Guttman split-half reliability was 0.610, and test-retest reliability was 0.644 (with an interval of 2 weeks)[18].

### Quality control

Before the start of the program, every member of the research team was given the same training regarding the aims, methods, and processes of the investigation. To increase the rate of participation, the details regarding the examination and questionnaire were explained to the parents and guardians of all children at a meeting prior to the examination. Before the survey, we pilot tested and refined the questionnaire’s language, logic, and sequence of questions. During the field data collection, the project members handed out all the questionnaires and gave detailed instructions on how to fill it in. An audit of the questionnaire was conducted to increase its validity, with verification procedures that included checks by class teachers, audits by quality control staff (senior post-graduate students from the School of Public Health of Fudan University), and phone reviews when necessary.

### Definition and statistical analysis

A database was established using Epidata3.1 and double data entry and logic verification were conducted. Then statistical analysis was performed using the Statistical Package for the Social Sciences (SPSS) 16.0 (SPSS Inc., Chicago, IL, USA).

SE was determined with the following equation: SE = spherical degree + 0.5 × cylinder degree. The AL/CR ratio was defined as the AL divided by the mean corneal radius of curvature. The elongation of AL, increase of AL/CR ratio, change in SE and incident myopia were used to reflect the myopic shifts. As the right eye was closely related to the left eye (Pearson coefficient: AL = 0.945, AL/CR = 0.850, SE = 0.924, *P* < 0.001), only data from the right eye were included in analyzing elongation of the AL, increase of the AL/CR ratio and change in the SE. Myopia was defined as SE ≤ -0.50 D. Children were determined to have incident myopia if classified as not myopic at baseline and myopic in either eye at one-year follow-up. The incidence of myopia was defined as the proportion of children who were not myopic (at risk) at baseline and who subsequently developed myopia in either eye during the one-year follow-up period.

The quantitative indicators are presented here as mean ± standard deviation (mean ± SD) and qualitative indicators are described in relative terms. Comparisons of quantitative indicators between different groups were checked with a χ^2^ test and comparisons of the mean values of all the indicators between different groups were checked with an independent sample t test or analysis of variance. First, we described the basic information of participating students at baseline and follow-up. Second, we described the characteristics of myopic shifts and near work related behaviors by grade level and gender and comparisons of the mean values of all the indicators between various grades and different genders were checked with analysis of variance and an independent sample t-test. Then, to examine the associations between elongation of the AL, increase of AL/CR ratio and change in SE and near work related behaviors, linear regression analysis was used, including univariate and multivariate analyses. Finally, we explored the association between near work related behaviors and incident myopia computing the unadjusted and adjusted incidence odds ratios and 95% confidence intervals [CI] through logistic regression analysis. In conducting a multivariable logistic regression model, we retained those variables that were identified as predictors of myopia” in the literature including parental myopia, student’s gender, student’s age, outdoor activity and near work time [19,20,21]. All CIs were 95%. *P* values were considered significant at the 0.05 level.

### Ethics statement

This study was approved by the Ethics Committee of the Shanghai General Hospital, and the research was conducted in accordance with the Declaration of Helsinki. The nature and possible consequences of the study were explained at each school. After the school principal agreed to participate, the details regarding the examination and questionnaire were explained to the parents and guardians of all children at a meeting prior to the examination, and written informed consent was obtained from each. The children provided verbal consent on the day of the examination and survey. Examination after cycloplegia was only performed in children whose parents and guardians had agreed to their participation for all examination items. Children whose parents and guardians had agreed to participation in all examination items, except for cycloplegic refraction, were given an examination without cycloplegia.

## Results

### Participant characteristics

The difference in the mean AL, corneal radius (CR), and AL/CR ratio values and age of students who completed cycloplegic refraction (2,871) were not statistically significantly different from those who did not undergo cycloplegic refraction (1,351) (AL: t = -0.376, *P* = 0.707; CR: t = -0.950, *P* = 0.342; AL/CR: t = 0.446, *P* = 0.656; age: t = -0.983, *P* = 0.326). Girls tended not to complete cycloplegic refraction (χ^2^ = 5.129, *P* = 0.024). The difference between students who completed cycloplegic refraction compared to those who did not undergo cycloplegic refraction at one-year follow up among participants who had completed cycloplegic refraction at baseline was also analyzed. The difference in the CR, AL/CR, and SE values at baseline, and age of students and the gender distribution of the students who completed cycloplegic refraction (1,957) were not statistically significantly different from those who had not undergone cycloplegic refraction (883)(CR: t = -1.700, *P* = 0.089; AL/CR: t = -1.488, *P* = 0.137; SE: t = 1.872, *P* = 0.061; age: t = -1.215, *P* = 0.224; gender: χ^2^ = 0.134, *P* = 0.714).

To analyze the characteristics of near work related behaviors and their associations with elongation of AL and increase of AL/CR, we used the baseline valid questionnaire results and the valid information for AL and AL/CR at baseline and follow-up (n = 4129). We used the baseline valid questionnaire results and valid information of SE at both baseline and follow-up (n = 1934) to analyze the association between near work related behaviors and changes in SE. In our study, 1,477 were non-myopic at baseline and their data was used to analyze the association between near work related behaviors and incident myopia.

Among the 4,129 students who were included in the analysis, 2,220 (53.8%) were boys and 1,909 (46.2%) were girls, aged 8.11 ± 1.17 years (range 6.08–13.17 years). The mean height was 129.51 ± 12.60 centimeters (cm), and the students grew taller as they aged during the year. About half of the parents were both non myopic (47.5%) and 17.5% of parents were myopes. The average time spent on near work was 2.95 ± 1.72 hours per day, which increased as the grade levels increased (grades 1 to 3 changed from 2.39 to 3.38 hours per day) and then decreased (grades 3 to 4 changed from 3.38 to 3.11 hours per day). The average time spent outdoors was 1.29 ± 1.00 hours per day, which decreased as the grade level increased (range, 1.22–1.32; Table 1).

**Table 1:** Participant characteristics.

Variable	n = 4,129 (Information from the valid questionnaire and valid information on AL and AL/CR at both baseline and follow-up)					n = 1,934 (Information from the valid questionnaire and valid information on SE at both baseline and follow-up)				
	Total	Grade 1	Grade 2	Grade 3	Grade 4	Total	Grade 1	Grade 2	Grade 3	Grade 4
n	4,129	1,095	1,117	1,063	854	1,934	543	535	468	388
Age (years)	8.11±1.17	6.67±0.33	7.70±0.37	8.73±0.42	9.71±0.45	8.08±1.20	6.67±0.33	7.70±0.39	8.73±0.44	9.79±0.47
Gender										
Boys	2,220 (53.8)	588 (53.7)	584 (52.3)	583 (54.8)	465 (54.4)	1,067 (55.2)	311 (57.3)	279 (52.1)	267 (57.1)	210 (54.1)
Girls	1,909 (46.2)	507 (46.3)	533 (47.7)	480 (45.2)	389 (45.6)	867 (44.8)	232 (42.7)	256 (47.9)	201 (42.9)	178 (45.9)
Height (cm)	129.51±12.60	122.13±6.48	127.16±7.80	131.82±17.73	139.16±8.17	129.50±11.41	121.82±7.59	127.08±7.88	132.48±13.65	139.98±6.88
Outdoor time (hours/day)	1.29±1.00	1.32±0.92	1.27±0.94	1.26±1.07	1.22±1.02	1.35±1.05	1.41±1.01	1.33±0.97	1.33±1.05	1.32±1.22
Near work time (hours/day)	2.95±1.72	2.39±1.59	2.89±1.54	3.38±1.88	3.11±1.53	3.00±1.74	2.54±1.67	3.03±1.71	3.38±1.89	3.12±1.53
Parental myopia										
Neither	1,772 (47.5)	461 (45.6)	510 (51.3)	434 (45.4)	367 (47.8)	935 (54.3)	270 (55.0)	267 (56.1)	219 (52.9)	179 (52.5)
Either	1,305 (35.0)	354 (35.0)	317 (31.9)	356 (37.2)	278 (36.2)	548 (31.8)	149 (30.3)	152 (31.9)	132 (31.9)	115 (33.7)
Both	652 (17.5)	197 (19.5)	167 (16.8)	166 (17.4)	122 (15.9)	239 (13.9)	72 (14.7)	57 (12.0)	63 (15.2)	47 (13.8)

### Characteristics of myopic shifts

From 2013 to 2014, the mean AL (23.15 ± 0.92 mm) had elongated by (0.32 ± 0.35 mm), the mean AL/CR ratio (2.96 ± 0.11) had increased by 0.032 ± 0.054, and the mean SE (0.36 ± 1.44 D) had changed by (-0.51) ± 0.51 D. The incidence of myopia in one year was 16.0% (237/1,477).

### The score of near work related behaviors by grade level

The scores of each special subcategory of near work related behaviors can be classified into four types according to grade level. The score for keeping a reasonable distance from the eye when reading, writing and watching TV, keeping a correct hand posture to hold a pen when writing and keeping correct body posture when reading and writing decreased at first (grades 1 to 2) and then increased (grades 2 to 4), as in the shape of a “U” (“U-Type”). Scores for not reading in situations that can lead to unstable body posture, selecting an adequate lighting environment for visual comfort when reading and writing and keeping a balanced diet increased at first (grades 1 to 2) and then decreased (grades 2 to 4), as in the shape of a “Inverted U” (“Inverted U Type”). The scores for finding various ways to have eye breaks and doing eye exercises according to the right way increased from grades 1 to 4. The scores for not continuing to do near work for more than 30–40 minutes without an eye break decreased from grades 1 to 4. Except for finding various ways to take eye breaks, keeping a reasonable distance when reading, writing and watching TV and keeping correct hand posture to hold a pen when writing, the differences between other special dimensions of near work related behaviors were significant between boys and girls. Boys did better regarding selecting an adequate lighting environment for visual comfort when reading and writing and keeping a balanced diet, while girls did better at not continuing to do near work for more than 30–40 minutes without an eye break, doing eye exercises according to the right way, not reading in situations that can lead to unstable body posture and keeping correct body posture when reading and writing (Table 2).

**Table 2:** Baseline near work related behaviors by grade level and gender.

Near work related behaviors	Total	Grade						Gender			
		Grade 1	Grade 2	Grade 3	Grade 4	F	P	Male	Female	t	P
Finding various ways of eye breaks	14.87±5.41	14.07±4.95	14.22±4.90	15.31±5.87	16.17±5.72	33.581	<0.001	14.74±5.43	15.02±5.39	-1.697	0.09
Not continuing to do near work for more than 30–40 minutes without eye break	9.11±1.92	9.25±1.91	9.10±1.81	9.06±1.93	9.02±2.06	2.922	0.033	9.03±1.91	9.21±1.93	-3.079	0.002
Doing eye exercises according to the right way	4.97±1.03	4.92±0.97	4.98±1.04	4.99±1.02	5.01±1.08	1.343	0.259	4.88±1.03	5.08±1.02	-6.073	<0.001
Keeping a reasonable distance with the eye when reading, writing and watching TV	15.55±2.83	15.21±2.67	15.11±2.75	15.73±2.93	16.32±2.82	38.697	<0.001	15.62±2.79	15.47±2.88	1.756	0.079
Keeping correct hand posture to hold a pen when writing	5.61±1.53	5.49±1.47	5.32±1.46	5.67±1.56	6.04±1.54	41.757	<0.001	5.59±1.52	5.62±1.53	-0.611	0.541
Keeping correct body posture when reading and writing	7.94±1.89	7.69±1.73	7.59±1.73	8.10±2.00	8.49±2.01	48.649	<0.001	7.86±1.88	8.02±1.90	-2.66	0.008
Not reading in situations that can lead to unstable body posture	3.37±2.35	3.32±2.12	3.43±2.25	3.36±2.45	3.36±2.63	0.474	0.7	3.46±2.43	3.26±2.25	2.82	0.005
Selecting an adequate lighting environment and having visual comfort when reading and writing	2.79±1.03	2.76±1.00	2.86±1.09	2.82±1.06	2.69±0.97	5.253	0.001	2.82±1.06	2.75±1.00	2.112	0.034
Keeping a balanced diet	4.59±1.54	4.76±1.53	4.77±1.49	4.53±1.55	4.20±1.54	29.661	<0.001	4.66±1.56	4.50±1.52	3.391	0.001

### Near work related behaviors and associations with elongation of axial length and increase of axial length/corneal radius

To investigate which special near work related behaviors were related to elongation of the AL and increase of AL/CR ratio, we carried out univariate and multivariate analyses for all the students. The results are shown in Table 3. In univariate analysis, only a single special dimension of near work related behaviors was included, while in multivariate analysis, all the listed covariates and each of the near work related behaviors (coded as continuous variables) were included. Keeping a reasonable distance when reading, writing and watching TV, selecting an adequate lighting environment for visual comfort when reading and writing were significantly related to elongation of the AL in multivariate analysis. The increase in AL was significantly associated with a low score for keeping a reasonable distance when reading, writing and watching TV [standardized coefficient beta = -0.062, *P* = 0.004] and a low score for selecting an adequate lighting environment for visual comfort when reading and writing [beta = -0.039, *P* = 0.034)].

**Table 3:** Near work related behaviors associated with elongation of axial length within one year in students of grade 1 to 4 in Shanghai.

Near work related behaviors	Elongation of Axial Length					
	Univariate analysis			Multivariate analysis		
	*t*	*P*	Standardized Coefficient Beta	*t*	*P*	Standardized Coefficient Beta
Finding various ways of eye breaks	**-2.027**	**0.043**	**-0.032**	-0.548	0.584	-0.011
Not continuing to do near work for more than 30–40 minutes without an eye break	**-1.919**	**0.055**	**-0.030**	-0.284	0.777	-0.005
Doing eye exercises according to the right way	1.743	0.081	0.027	0.462	0.644	0.008
Keeping a reasonable distance when reading, writing and watching TV	**-3.284**	**0.001**	**-0.051**	**-2.881**	**0.004**	**-0.062**
Keeping a correct hand posture to hold a pen when writing	-0.979	0.328	-0.015	0.610	0.542	0.012
Keeping correct body posture when reading and writing	-0.764	0.445	-0.012	0.915	0.360	0.019
Not reading in situations that can lead to unstable body posture	-0.143	0.887	-0.002	-0.769	0.442	-0.015
Selecting an adequate lighting environment and having visual comfort when reading and writing	**-2.916**	**0.04**	**-0.045**	**-2.127**	**0.034**	**-0.039**
Keeping a balanced diet	-1.963	0.050	-0.031	-1.228	0.220	-0.022
Parental myopia						
Neither	-	-	-	-	-	-
Either	**2.432**	**0.015**	**0.044**	1.437	0.151	0.027
Both	**5.855**	**<0.001**	**0.106**	**5.291**	**<0.001**	**0.098**
Age	**-2.372**	**0.018**	**-0.037**	**-2.638**	**0.008**	**-0.052**
Gender						
Boy	-	-	-	-	-	-
Girl	2.007	0.045	0.031	1.286	0.198	0.022
height	0.046	0.964	0.001	0.985	0.325	0.019
Near work time	-1.157	0.247	-0.019	0.313	0.765	0.005
Outdoor time	**-3.577**	**<0.001**	**-0.058**	**-2.227**	**0.026**	**-0.039**

Not continuing to do near work for more than 30–40 minutes without an eye break and selecting an adequate lighting environment for visual comfort when reading and writing were significantly related to increase of the AL/CR ratio in multivariate analysis. The increase in the AL/CR ratio was significantly associated with low scores for not continuing to do near work for more than 30–40 minutes without an eye break [beta = -0.028, *P* = 0.044] and low scores for selecting an adequate lighting environment for visual comfort for reading and writing [beta = -0.030, *P* = 0.048]. (Table 4)

**Table 4:** Near work related behaviors associated with axial increase of length/corneal curvature within one year in students of grade 1 to 4 in Shanghai.

Near work related behaviors	Increase of Axial Length/Corneal Curvature					
	Univariate analysis			Multivariate analysis		
	*t*	*P*	Standardized Coefficient Beta	*t*	*P*	Standardized Coefficient Beta
Finding various ways of eye breaks	-0.84	0.401	-0.013	-0.138	0.890	-0.003
Not continuing to do near work for more than 30–40 minutes without an eye break	**-2.890**	**0.004**	**-0.024**	**-2.020**	**0.044**	**-0.028**
Doing eye exercises according to the right way	1.518	0.129	0.045	1.926	0.054	0.033
Keeping a reasonable distance when reading, writing and watching TV	-1.146	0.252	-0.018	-0.963	0.336	-0.021
Keeping a correct hand posture to hold a pen when writing	-1.310	0.190	-0.020	-0.715	0.475	-0.014
Keeping correct body posture when reading and writing	0.195	0.845	0.003	1.108	0.268	0.023
Not reading in situations that can lead to unstable body posture	-1.222	0.222	-0.019	-1.501	0.133	-0.029
Selecting an adequate lighting environment and having visual comfort when reading and writing	**-2.760**	**0.006**	**-0.043**	**-1.972**	**0.048**	**-0.030**
Keeping a balanced diet	**-3.082**	**0.002**	**-0.048**	-1.248	0.212	-0.023
Parental myopia						
Neither	-	-	-	-	-	-
Either	**2.627**	**0.009**	**0.048**	1.481	0.139	0.028
Both	**4.013**	**<0.001**	**0.073**	**3.608**	**0.000**	**0.067**
Age	-0.967	0.333	-0.015	-1.304	0.192	-0.026
Gender						
Boy	-	-	-	-	-	-
Girl	1.467	0.142	0.023	0.617	0.537	0.010
height				0.630	0.529	0.012
Near work time	-0.904	0.366	-0.015	0.088	0.930	0.002
Outdoor time	**-3.421**	**0.001**	**-0.055**	**-2.15**	**0.031**	**-0.038**

### Near work related behaviors and associations with change in refractive error and incident myopia

Not continuing to do near work for more than 30–40 minutes without an eye break and keeping a reasonable distance when reading, writing and watching TV were significantly related to changes in SE in the multivariate analysis. The decrease in SE was significantly associated with low scores for not continuing to do near work for more than 30–40 minutes without an eye break [beta = -0.064, *P* = 0.023] and low scores for keeping a reasonable distance when reading, writing and watching TV [beta = -0.072, *P* = 0.020]. (Table 5)

**Table 5:** Near work related behaviors associated with changes in refractive error within one year in students of grades 1 to 4 in Shanghai.

Near work related behaviors	Changes in refractive error					
	Univariate analysis			Multivariate analysis		
	*t*	*P*	Standardized Coefficient Beta	*t*	*P*	Standardized Coefficient Beta
Finding various ways of eye breaks	-0.758	0.448	**-0.017**	0.496	0.620	0.015
Not continuing to do near work for more than 30–40 minutes without an eye break	**-2.188**	**0.029**	**-0.050**	**-2.280**	**0.023**	**-0.064**
Doing eye exercises according to the right way	-0.377	0.706	-0.009	-1.600	0.110	-0.042
Keeping a reasonable distance when reading, writing and watching TV	**-2.401**	**0.016**	**-0.055**	**-2.331**	**0.020**	**-0.072**
Keeping a correct hand posture to hold a pen when writing	0.401	0.688	0.009	1.728	0.084	0.048
Keeping correct body posture when reading and writing	-0.348	0.728	0.006	-0.553	0.580	-0.016
Not reading in situations that can lead to unstable body posture	-0.428	0.669	-0.010	-1.469	0.142	-0.009
Selecting an adequate lighting environment and having visual comfort when reading and writing	**-2.549**	**0.011**	**-0.058**	-1.101	0.271	-0.029
Keeping a balanced diet	**-3.074**	**0.002**	**-0.070**	-1.012	0.312	-0.028
Parental myopia						
Neither	-	-	-	-	-	-
Either	**4.041**	**<0.001**	**0.107**	**2.570**	**0.010**	**0.105**
Both	**3.344**	**0.001**	**0.088**	**3.670**	**<0.001**	**0.096**
Age	**2.430**	**0.015**	**0.055**	1.863	0.063	0.050
Gender						
Boy	-	-	-	-	-	-
Girl	**3.107**	**0.002**	**0.071**	**2.570**	**0.010**	**0.066**
Near work time	0.273	0.785	0.006	0.883	0.377	0.023
Outdoor time	**-2.917**	**0.004**	**-0.069**	**-2.117**	**0.034**	**-0.057**

To investigate which special near work related behaviors were related to incident myopia, we carried out univariate and multivariate logistic regression analyses The results are shown in Table 6. Incident myopia was regarded as the dependent variable and each dimension of near work related behaviors was treated as an independent variable. Incident myopia was significantly associated with low scores for keeping a reasonable distance when reading, writing, and watching TV (adjusted odds ratio [aOR] = 0.90, 95%CI: 0.84–0.96).

**Table 6:** Near work related behaviors associated with incident myopia within one year in students of grades 1 to 4 in Shanghai.

Near work related behaviors	Incident myopia (n = 1,477)			
	Univariate analysis		Multivariate analysis	
	cOR	95%CI	aOR	95%CI
Finding various ways to take eye breaks	0.99	0.91–1.02	1.00	0.97–1.04
Not continuing to do near work for more than 30–40 minutes without an eye break	0.97	0.90–1.04	0.97	0.89–1.06
Doing eye exercises according to the right way	0.97	0.85–1.12	0.89	0.76–1.04
Keeping a reasonable distance when reading, writing and watching TV	**0.94**	0.90–0.99*	**0.90**	0.84–0.96*
Keeping a correct hand posture to hold a pen when writing	0.99	0.91–1.09	1.03	0.92–1.15
Keeping correct body posture when reading and writing	1.01	0.94–1.09	1.05	0.94–1.16
Not reading in situations that can lead to an unstable body posture	0.98	0.93–1.04	1.03	0.96–1.11
Selecting an adequate lighting environment and having visual comfort when reading and writing	0.93	0.81–1.06	0.86	0.73–1.02
Keeping a balanced diet	1.07	0.98–1.17	1.04	0.93–1.16
Parental myopia				
Neither	-	-	1.00	-
Either	1.30	0.92–1.83	1.40	0.97–2.01
Both	**2.25**	**1.46–3.45**	**2.49**	**1.56–3.97**
Age	**1.25**	**1.11–1.40**	**1.27**	**1.10–1.46**
Gender				
Boy	-	-	1.00	-
Girl	1.01	0.76–1.35	1.07	0.78–1.47
Near work time	1.07	0.98–1.16	1.05	0.96–1.16
Outdoor time	1.02	0.88–1.17	1.08	0.92–1.27

Note; cOR: crude odds ratio; aOR: adjusted odds ratio;

* *P*<0.05

## Discussion

In our longitudinal study on school students in Shanghai, primary students’ near work related behaviors changed by grade level at baseline: the score of some subcategories increased/decreased from grades 1 to 4 and others increased/decreased at first (grades 1 to 2) and then decreased/increased (grades 2 to 4). A change in myopia related oculometric parameters, such as AL, AL/CR ratio, SE, and incident myopia, were significantly associated with some special dimensions of near work related behaviors. Keeping an inappropriate distance when reading, writing and watching TV, selecting an inadequate lighting environment and having visual discomfort when reading and writing and continuing to do near work for more than 30–40 minutes without an eye break were risk factors for myopic shifts.

In our study, changes in various near work related behaviors by grade level were explored. Each subcategory of near work related behaviors presented various characteristics. Primary student’s near work related behaviors can be classified into four changing types according to grade level. The scores for keeping a reasonable distance when reading, writing and watching TV, keeping a correct hand posture to hold a pen when writing and keeping correct body posture when reading and writing showing as a “U-type” meant the behavior was better at the lower grade level (grade 1) and higher grade level (grade 4). The behaviors being better in grade 1 may be the result of education, since teachers always give instructions on how to develop good habits for reading and writing when primary students enter school. Those three near work related behaviors being better in grade 4 may due to the rich knowledge and improved cognitive ability with growth [22], which was also applied to finding various ways of taking eye breaks and doing eye exercises according to the right way increasing from grades 1 to 4. Scores for not reading in situations that can lead to unstable body posture, selecting an adequate lighting environment for visual comfort when reading and writing and keeping a balanced diet increased at first (from grades 1 to 2) and then decreased (from grades 2 to 3) and these behaviors were best in grade 2. The reason may be the combination of education and imbalance in behavior and cognitive ability [23]. The scores for not continuing to do near work for more than 30–40 minutes without an eye break decreased as the grade level increased, which may due to increased academic load [24].

In this study, the mean elongation of axial length was 0.32 ± 0.35 mm and the mean increase in the AL/CR ratio was 0.032 ± 0.054. These data were comparable with the results obtained in the study by Guo et al. who found a mean elongation of 0.26 ± 0.49 for AL and 0.03 ± 0.06 for AL/CC in primary students in Beijing, China [25]. In a similar study conducted in Zhongshan Ophthalmic Centre, Donovan and colleagues found that mean axial elongation was 0.17 ± 0.10 mm for summer, 0.24 ± 0.09 mm for autumn, 0.24 ± 0.09 mm for winter, and 0.15± 0.08 mm for spring[26]. The mean change in SE in our study was -0.51 ± 0.51 D, which was comparable with other studies. Among Northern Irish schoolchildren, the mean change in SER was -0.38 D [interquartile range -0.75 D to 0 D] for 6-to7-year-olds and -0.13 D (interquartile range -0.38 D to 0.25 D) for 12- to 13-year-olds, respectively, in 3 years [27]. Previous studies focused on myopic shifts in Chinese students mainly used indicators of noncycloplegic autorefraction. Wu et al. reported the mean refractive error -1.13 ± 1.57 D had changed -0.52 ± 0.73 D from baseline (2010) to follow-up (2011) with a noncycloplegic autorefraction examination in 6–12 year old students in Beijing, China [14]. The incidence of myopia in our study was 16.0%, and grew as the age increased overall. Amanda et al. reported the 5 to 6 year cumulative incidence of myopia was 14.8% for 6-year-old children and 17.3% for 12-year-old in The Sydney Adolescent Vascular and Eye Study. In our study, the one year incidence of 6-year-old students was 12.8%, which was higher than that of Australian schoolchildren (annual incidence of 2.4%). What is more, the incidence of 9-year-olds was 23.4%, which was also higher than that of Australian schoolchildren aged 12 years (annual incidence of 3.8%)[8].

In our study, special near work related behaviors including keeping an inappropriate distance when reading, writing and watching TV, selecting an inadequate lighting environment for visual discomfort when reading and writing and continuing to do near work for more than 30–40 minutes without an eye break were associated with shifts towards myopia. These findings were consistent with some studies [12,13,28]. The findings in Australian school children who performed near-work at a distance of less than 30 cm were more likely to have myopia than those who worked at a longer distance [12,29]. A similar finding was reported on primary school children in Beijing, China, which concluded that shorter reading distances were associated with greater shifts towards myopia in univariate analysis [14]. Yang and colleagues explored the effect of different reading distances on the accommodation of children and found accommodative lag existed for near distance. Accommodative lag increased with decreasing reading distances. Accommodative lag may induce hyperopic retinal defocus, which may promote myopia progression [13,29]. A previous study by Rose et al. suggested that bright outdoor light might prevent myopia by increasing the release of dopamine from the retina, since dopamine has been known to be an inhibitor of axial elongation [30,31]. In an animal model such as chickens, bright light has been shown to prevent the development of myopia and this prevention was dopamine-dependent [32,33]. That may be the possible reason for the effect of selecting an inadequate lighting environment and having visual discomfort when reading and writing on myopic shifts. A similar finding was also reported by You et al. in that dim reading illumination was associated with the prevalence of myopia [7]. Hua et al. suggested elevated light levels in classrooms have a significant effect on myopia onset, decreases in refraction, and axial growth in an intervention study [34]. The finding of an association between continuing to do near work for more than 30–40 minutes without an eye break and myopic shifts was consistent in a study on epidemiological characteristics of vision care and an intervention study in Shanghai primary schools conducted by Tan et al. In that study, spherical degrees without cycloplegic autorefraction were associated with continuing to do near work for more than 30–40 minutes without an eye break in canonical correlation analysis [23]. Similar findings were also seen in The Beijing Childhood Eye Study [7]. However, some researches didn’t find above associations. Wu LJ reported shorter reading distance wasn’t independently associated with myopic shift in multivariate analysis after controlling potential factors [14]. In the Anyang Childhood Eye Study, Li SM et al reported reading distance (≤ 20cm) was associated with SE but not with presence of myopia [34]. The difference may lie in the measuring method of behaviors and various environments included. Some studies just used only one question to measure one near work related behavior. For example, Ip JM used the distance from book to face to reflect reading distance [12]. Other studies used questionnaire to collect near work related behaviors without evaluating the questionnaire [14]. What’s more, some studies have only focused on one special behavior related to near work, others focused on several related behaviors together with outdoor activities and near work time [7,14]. Near work related behaviors have not been researched fully. In this study, comprehensive and multi-dimensional near work related behaviors were fully analyzed. We used the CVCBS to get information of near work related behaviors with acceptable reliability and validity. Moreover, different outcomes were used including biometry(AL and AL/CR ratio) and cycloplegic refraction (SE and incident myopia) and similar effects were found.

The characteristics of the above three near work related behaviors associated with myopic shift were varied; i.e., keeping a reasonable distance when reading, writing and watching TV presented as a “U-type,” selecting an adequate lighting environment for visual comfort when reading and writing presented as an “Inverted U Type” and not continuing to do near work for more than 30–40 minutes without an eye break decreased as the grade level increased. As near work related behaviors were modifiable and had effects on myopic shifts, it was better to suggest targeted intervention and focus on specific students through education to promote students developing good behaviors to prevent myopia.

The results of our study suggested a new perspective on myopia prevention through comprehensive understanding of the characteristics of near work related behaviors. Since near work related behaviors accompany every student when they study, they do not need extra time and it is easy to get support from school teachers and parents. Moreover, near work related behaviors can be improved through education. Future studies may focus on keeping a reasonable distance when reading, writing, and watching TV, selecting an adequate lighting environment for visual comfort when reading and writing and not continuing to do near work for more than 30–40 minutes without an eye break, and an interventional study should be designed to explore preventing myopia.

This study was a school-based one-year cohort study focused on near work related behaviors and their associations with myopic shifts. The outcomes used in analysis included both biometry and cycloplegic refraction, which was rarely reported before, especially in China. We also presented the effect of near work related behaviors on myopic shifts in all the participating students, students completing cycloplegic refraction both at baseline and follow-up and non-myopic students at baseline. This study was the first to comprehensively and multi-dimensionally explore near work related behaviors and associations with myopic shifts. The study was conducted rigorously using unified equipment and a standardized examination process, implementation by experienced public health physicians and collecting data with an entire process of quality control. Eye physicians, data collectors, as well as quality control and other personnel, received training prior to the study. The questionnaire was pilot tested before it was used in fieldwork and its validity and reliability were tested.

Nonetheless, the study had some potential limitations. First, the data on the near work related behaviors were self-reported so that estimates of near work related behaviors could be subject to recall bias. An effort was made to reduce recall bias by inquiring about the information of the previous week and asking both students and their parents or guardians. Second, the score of students’ near work related behaviors changed by grade level as reported. Therefore, it is hard to value whether the behavior was unchangeable during the one-year follow-up. However, we conducted the study from September 2013 to October 2014. In fact, all the eye examinations were started from September to October at both baseline (2013) and follow-up (2014) and the questionnaire survey was implemented in September 2013. The period of a grade level starts from September to July of the next year. A student’s academic load, curriculum design, and teachers change little at the same grade level. Therefore, near work related behaviors at baseline can be representative of the whole year. In a future study, it would be better to perform the questionnaire survey at both baseline and follow-up. Third, another possible limitation was the use of subjective measures for near work related behaviors with answers of frequency. However, such methods have also been used in another previous study [35].

## Conclusion

In conclusion, various near work related behaviors would change according to grade in primary students. Independent of hereditary factors, the daily near work time and outdoor activity time, keeping an inappropriate distance with the eye when reading, writing and watching TV, selecting an inadequate lighting environment for visual discomfort when reading and writing and continuing to do near work for more than 30–40 minutes without an eye break were associated with a change in oculometric parameters with both biometry and cycloplegic refraction indicating an increase in myopia in Shanghai within a study period of one year. Our longitudinal study provided additional information on the potentially protective role of near work related behaviors on the myopic change towards myopia. Public health care measures are necessary such as that school teachers and parents take account of the potential for urging students to develop good near work related behaviors to prevent the onset and progression of myopia. Future intervention studies may also potentially address near work related behaviors especially for keeping a reasonable distance, selecting an adequate lighting environment and not continuing to do near work for more than 30–40 minutes without an eye break.

## Supporting Information

S1 File Content of The Child Vision Care Behaviors Scale. (DOCX)
