# Supplementary material for: Near Work Related Behaviors Associated with Myopic Shifts among Primary School Students in the Jiading District of Shanghai: A School-Based One-Year Cohort Study
Source: PLoS One. 2016 May 3;11(5):e0154671. doi: 10.1371/journal.pone.0154671 (PMC4854402; doi:10.1371/journal.pone.0154671)
Supplement: S1 File — (DOCX) [file pone.0154671.s001.docx]

**Content of The Child Vision Care Behaviors Scale (CVCBS)**

| Subscales | Items |
| --- | --- |
| Finding various ways to take eye breaks | After 30-40 minutes reading and writing in near distance,  1) Taking eye breaks by looking far into the distance  2) Taking eye breaks by doing light physical movement  3) Taking eye breaks by taking turns to looking far and near into the distance  4) Taking eye breaks by doing eye exercises (Chinese eye acupuncture point massage)  5) Taking eye breaks by sitting in repose with eyes closed  6) Taking eye breaks by doing outdoor activities  7) Taking eye breaks by looking at green plants |
| Not continuing to do near work for more than 30~40 minutes without eye break | 1) Continuing reading and writing for more than 30~40 minutes without eye break (recode)  2) Continuing watching TV for more than 30~40 minutes without eye break  3) Continuing playing with the computer for more than 30~40 minutes without eye break |
| Doing eye exercises according to the right way | 1) Having acid bilge feeling when doing eye exercises  2) Doing eye exercises with incorrect methods such as opening the eyes, lacking of concentration (recode) |
| Keeping the reasonable distance with the eye when reading, writing and watching TV | 1) The distance between eyes and books is less than 30 cm (recode)  2) The distance between eyes and TV is less than 2 m (recode)  3) The distance between eyes and computer screen is less than 50 cm (recode)  **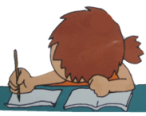**4) Bending over the desk when doing the homework (recode)  5) Writing with head tilted (recode) |
| Keeping correct hand posture to hold pen when writing | **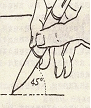**1) Keeping correct hand posture to hold pen and writing at 45 angle degree of the exercise book  2) Writing with the fingertip too close to the nib (<3cm) (recode) |
| Keeping correct body posture when reading and writing | 1) Writing with spine straight and keeping a proper distance between the chest and the desk  2) Writing with hips and thighs on the chair and feet on the ground or footboards  3) Writing with part of the hips away from the chair and chest close to the desk (recode) |
| Not reading in situations that can lead to unstable body posture | 1) Reading or playing with iPad when lying face up/down on the bed (recode)  2) Watching TV when lying face up/down on the bed (recode)  3) Playing with the mobile phone or iPad in the quilt (recode)  4) Reading or playing with the mobile phone or iPad in shaking vehicles (recode)  5) Walking when reading or playing with the mobile phone (recode)  6) Having meals when reading or playing with the mobile phone or iPad (recode) |
| Selecting environment of light adequate and visual comfort to read and write | 1) Reading and writing under adequate light.  2) Playing with mobile phone or iPad in the dark (recode) |
| keeping a balanced diet | 1) Having supplements of two servings of fruits and two servings of vegetables (100g=1 serving)  2) Keeping a balanced diet |
